# Left atrioventricular coupling index in patients undergoing cardiac resynchronization therapy

**DOI:** 10.1007/s10554-026-03641-9

**Published:** 2026-02-14

**Authors:** Cheyenne S. L. Chiu, Willem Gerrits, Sophie C. Rier, Philippe C. Wouters, Maarten J. Cramer, Ivo A. C. van der Bilt, Pim van der Harst, Muhammed I. Girgin, Kevin Vernooy, Antonius M. W. van Stipdonk, Vokko P. van Halm, Vincent F. van Dijk, Abdul Ghani, Alexander H. Maass, Sing C. Yap, Frebus J. van Slochteren, Mathias Meine, Marco Guglielmo

**Affiliations:** 1 Department of Cardiology, Division Heart & Lungs, University Medical Center Utrecht, Utrecht, The Netherlands; 2 Department of Cardiology, Haga Teaching Hospital, The Hague, The Netherlands; 3 Faculty of Medicine, Yıldırım Beyazıt University, Ankara, Türkiye; 4 Department of Cardiology, Cardiovascular Research Institute Maastricht (CARIM), University Medical Center+, Maastricht, The Netherlands; 5 Department of Cardiology, Amsterdam University Medical Center, Amsterdam, The Netherlands; 6 Department of Cardiology, St. Antonius Hospital, Nieuwegein, The Netherlands; 7 Department of Cardiology, Isala Heart Center, Zwolle, The Netherlands; 8 Department of Cardiology, UMCG Heart Center, University Medical Center Groningen, Groningen, The Netherlands; 9 Department of Cardiology, Cardiovascular Institute, Erasmus MC, Thorax center, Rotterdam, The Netherlands; 10 CART-Tech BV, Utrecht, The Netherlands; 11 Department of Radiology, University Medical Center Utrecht, Utrecht, The Netherlands

**Keywords:** Left atrioventricular coupling index, Cardiac resynchronization therapy, Echocardiography, Imaging, Heart failure

## Abstract

The left atrioventricular coupling index (LACI), defined as the ratio of left atrial (LA) to left ventricular (LV) end-diastolic volumes, serves as a surrogate for atrioventricular coupling. Cardiac resynchronization therapy (CRT) promotes reverse remodeling of both chambers and may enhance atrioventricular coupling, yet the clinical utility of LACI in CRT patients remains unclear. This study evaluated the effect of CRT on LACI and assessed its prognostic value for CRT response. In 123 patients enrolled in the ADVISE-CRT III trial (mean age 66 ± 10 years; 63% male; and 81% with a Class I CRT indication), LACI was measured by echocardiography before and six months after CRT implantation. Multivariable linear and logistic regression analyses were performed to assess the association between LACI and CRT response (≥ 15% LV end-systolic volume reduction), adjusting for potential confounders. Low baseline LACI was independently associated with an 11.2% greater LV end-systolic volume reduction (Quartile 1–2 vs. Quartile 3–4; *p* = 0.009) and 3.6-fold higher odds of CRT response (*p* = 0.041). At six months, LACI increased significantly in responders (16.5% to 20.7%; *p* < 0.001), driven by greater LV versus LA end-diastolic volume reduction. In non-responders, LACI remained stable (20.9% to 20.3%; *p* = 0.077). LACI was independently associated with CRT response and provided incremental value over individual LA and LV volumetric parameters. While LACI may reflect favorable structural substrate and atrioventricular coupling at baseline, it is not suitable for post-CRT assessment of atrioventricular coupling, as disproportionate LV versus LA remodeling may conceal coupling improvements.

**Graphical abstract:**

**Figure:**
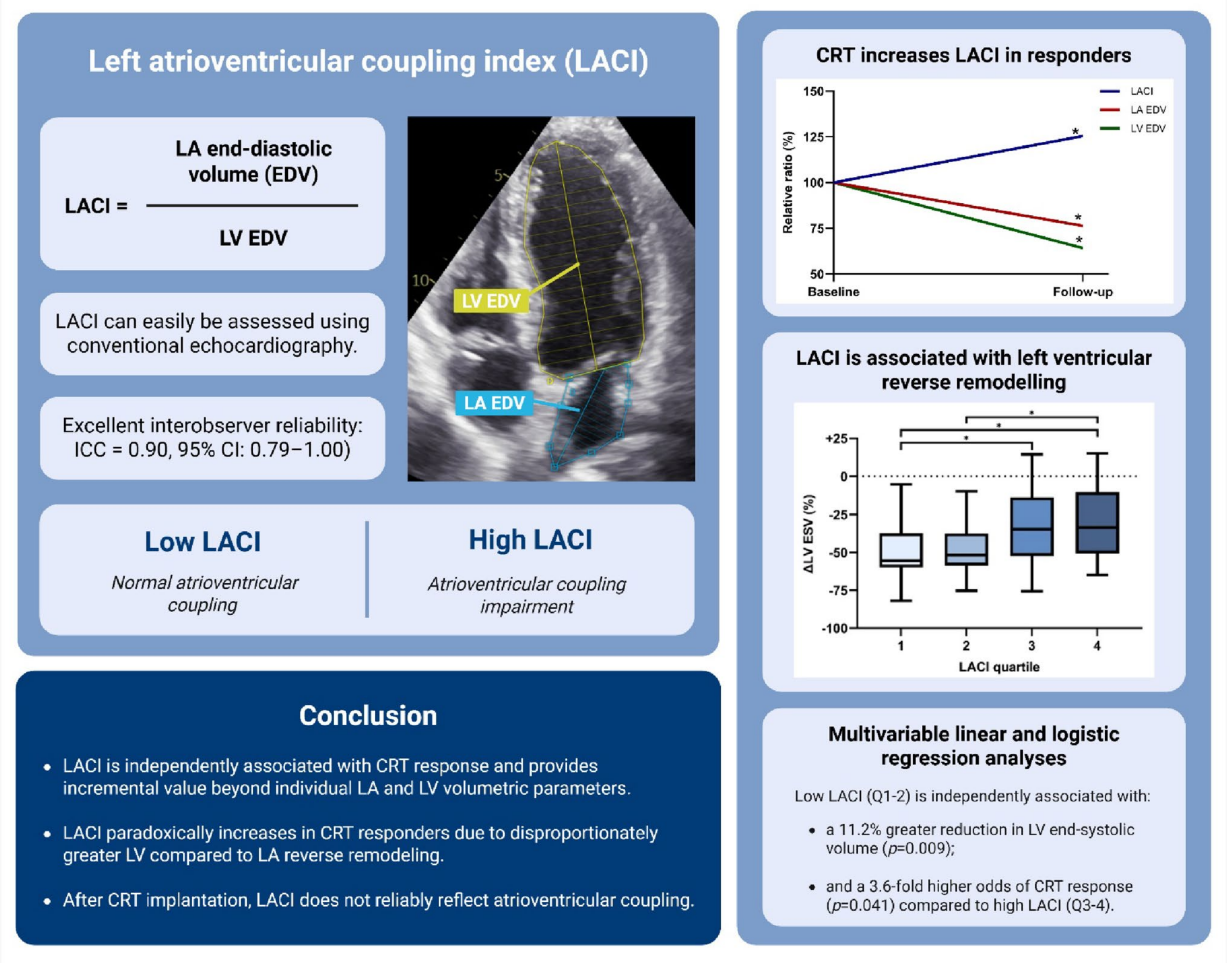
Left atrioventricular coupling index in patients undergoing cardiac resynchronization therapy

**Supplementary Information:**

The online version contains supplementary material available at 10.1007/s10554-026-03641-9.

## Introduction

Cardiac resynchronization therapy (CRT) is a well-established treatment for heart failure patients with ventricular dyssynchrony and reduced left ventricular (LV) ejection fraction despite optimal medical therapy [1, 2]. The beneficial effect of CRT in patients with a left bundle branch block (LBBB) is mainly attributed to inter- and intraventricular resynchronization. In patients with a wide QRS interval but non-LBBB morphology however, the extent to which ventricular resynchronization can be achieved remains ambiguous [3]. Therefore, alternative mechanisms, alongside ventricular resynchronization, may play a role in mediating the benefits of CRT in these patients [4].

High-grade intraventricular conduction delays are often accompanied by atrioventricular (AV) conduction disturbances, with prolonged PR intervals in 18–52% of CRT patients [5]. These conduction delays may result in AV uncoupling, elevated LV end-diastolic pressure, diastolic mitral regurgitation, and reduced LV pump function [4]. Prior research has demonstrated that patients with a prolonged PR interval experience greater benefits from CRT in terms of clinical outcomes [6–8], and that optimizing AV delay substantially enhances hemodynamics [9, 10]. This suggests CRT not only targets ventricular resynchronization but also restores AV coupling [5, 11]. A computational-experimental-clinical study using CircAdapt, an in-silico model of the human heart and circulation, supports this hypothesis by showing that biventricular pacing significantly improves hemodynamics in non-LBBB patients with a PR interval > 230 ms by restoring AV coupling [12]. However, the PR interval captures electrical AV conduction but not mechanical AV synchrony. Given the asynchronous interplay between the left atrium (LA) and LV, an integrated parameter assessing LA and LV simultaneously may offer a more physiologically comprehensive evaluation than conventional isolated measures. Traditionally, CRT response has been assessed through LV remodeling. However, CRT induces both LA and LV reverse remodeling [13, 14]. Growing evidence demonstrates that LA remodeling also predicts clinical outcomes [15–17], reinforcing the need for a more comprehensive assessment that includes both the LA and LV.

Recently, the left atrioventricular coupling index (LACI) has been introduced as a novel imaging metric. Defined as the ratio of LA to LV end-diastolic volume (EDV), LACI reflects AV coupling, with lower values indicating better AV function [18]. In heart failure patients, LACI has been shown to reflect the severity of diastolic dysfunction and to be independently associated with major adverse cardiovascular events, all-cause mortality and cardiovascular mortality [19–21].

While CRT may improve AV coupling and has been demonstrated to promote both LA and LV reverse remodeling, LACI has not yet been studied within the context of CRT patients. Our aim was therefore to evaluate the effect of biventricular pacing on LACI in CRT patients and to determine its prognostic value for CRT response.

## Methods

### Study population

The Advanced Image Supported Left Ventricular Lead Placement in Cardiac Resynchronization Therapy (ADVISE-CRT III) study was a randomized controlled trial, primarily designed to study on-screen image-guided lead placement in CRT patients. The study design of ADVISE-CRT III has been described in detail previously [22]. In brief, patients with a class I or IIa indication for CRT implantation were recruited across seven centers in the Netherlands. Participants were excluded if they had CMR contraindications, atrial fibrillation during CMR, or an eGFR of less than 30 ml/min/1.73m^2^. Patients were randomized to either the control group (conventional CRT implantation) or active group (on-screen image-guided CRT implantation). Stratified randomization based on ischemic cardiomyopathy ensured group balance.

Between February 2021 and October 2023, 131 patients from seven hospitals in the Netherlands were enrolled in the ADVISE-CRT III trial. Three patients were excluded due to an LV ejection fraction > 35%, one patient withdrew consent, and echocardiography at baseline or follow-up was of insufficient quality in four patients, resulting in a final sample of 123 patients. The process of inclusion, randomization and follow-up has been described in detail previously [23]. Six months post-implantation, CRT response was evaluated, defined as a reduction of LV end-systolic volume (ESV) ≥ 15%. For the present study, we divided the sample into responders and non-responders regardless of randomization outcome to investigate the effect of CRT on LACI.

This study was conducted in accordance with the Declaration of Helsinki. Ethical approval was obtained from the institutional review boards of all participating centers, and written informed consent was obtained from all participants. The trial is registered at clinicaltrials.gov under study ID NCT05053568.

### Echocardiography acquisition and analysis

All patients underwent comprehensive transthoracic echocardiography within three months preceding implantation and a follow-up echocardiogram six months after implantation. Two-dimensional, color, and continuous- and pulsed-wave Doppler images were acquired from the parasternal, apical, and subcostal views. LA and LV end-diastolic and end-systolic volumes and LV ejection fraction were measured using the biplane method of disks (modified Simpson) [24]. Volumes were then indexed to body surface area (BSA), applying the Mosteller formula [25].

Diastolic function was graded using the American Society of Echocardiography (ASE)/European Association of Cardiovascular Imaging (EACVI) algorithm for patients with depressed LV ejection fraction or myocardial disease [26]. Accordingly, the peak early (E) and atrial (A) velocities of mitral inflow and their ratio (E/A) were assessed using pulsed-wave Doppler. The lateral and septal mitral annular early diastolic velocities (e’) were measured and averaged. Subsequently, the ratio between the peak early transmitral velocity (E) and averaged mitral annular velocities e’ were calculated (E/e’) as an indicator for mean LV filling pressure. Valve insufficiencies, including tricuspid regurgitation velocity, were evaluated with continuous-wave Doppler and color images. Lastly, apical rocking, septal flash and interventricular mechanical delay were analyzed to evaluate inter- and intraventricular dyssynchrony [27].

Analysis was performed by a dedicated echocardiography core laboratory. All images were reviewed by an expert reader (M.G.) with more than a decade of experience in echocardiography and certified in adult echocardiography according to the standards of the European Society of Cardiology (ESC). To assess interobserver reliability, LACI was reanalyzed in 25 randomly selected echocardiograms by a blinded second expert reader (S.C.R.). Interobserver reliability was quantified using the Intraclass Correlation Coefficient (ICC). In case of disagreement, a third experienced operator (I.v.d.B.) was consulted to reach consensus.

### Left atrioventricular coupling index

LACI was calculated as the ratio of LA to LV EDV [18]. LA and LV volumes were measured during the end-diastolic phase around the closure of the mitral valve, when the LA reached its minimum volume and LV its maximum volume. LACI values are expressed as percentages, with higher values reflecting greater imbalance between LA and LV EDVs, indicating more severe impairment of left AV coupling [18].

### Statistical analysis

Normally distributed continuous data are reported as means ± standard deviation, and non-normally distributed data as medians with [interquartile range]. Categorical variables are presented as frequencies and percentages. Between-group differences were assessed using the Chi-square test, Mann-Whitney U test, and independent t-test as appropriate. Pairwise comparisons were conducted using the Kruskal–Wallis test followed by Dunn’s post hoc test with correction for multiple comparisons. Spearman’s rho (ρ) was used to assess correlations between variables. The incremental prognostic value of LACI beyond individual LA and LV volumetric parameters was examined using nested linear regression models. Prognostic performance of LACI alone was further compared with a model including both LA and LV EDVi using parallel models. Univariable and multivariable linear and logistic regressions were subsequently applied to assess associations with continuous (ΔLV ESVi) and binary (CRT response) outcomes. Potential confounders were included based on a *p*-value < 0.05 in univariable analyses. Assumptions of multivariable regression analyses were checked for the existence of non-linearity, heteroscedasticity, and multicollinearity by graphical analyses and correlations tests. All tests were performed two-tailed, with *p*-values < 0.05 considered significant. Analyses were conducted with SPSS (IBM Corp. Armonk, NY, USA, version 29.0.1).

**Table 1:** Baseline characteristics

	Total (*n* = 123)	Non-responders (*n* = 19)	Responders (*n* = 104)	*P*-value
*Demographics*				
Male sex	77 (62.6)	14 (73.7)	60 (57.7)	0.222
Age at implantation, years	65.7 ± 9.5	65.8 ± 7.8	65.7 ± 9.8	0.961
*Medical history*				
**New York Heart Association Class**				0.690
NYHA I	2 (1.6)	0 (0.0)	2 (1.9)	
NYHA II	88 (71.5)	13 (68.4)	75 (72.1)	
NYHA III	31 (25.2)	6 (31.6)	25 (24.0)	
Ischemic cardiomyopathy	49 (39.8)	11 (57.9)	38 (36.5)	0.092
Scar on CMR	58 (47.2)	13 (68.4)	45 (43.3)	0.052
Diabetes Mellitus (type 1/2)	23 (18.7)	4 (21.1)	19 (18.3)	0.99
Hypertension	52 (42.3)	10 (52.6)	42 (40.4)	0.636
History of smoking	45 (36.6)	9 (47.4)	36 (34.6)	0.552
Percutaneous Coronary Intervention	27 (22.0)	2 (10.5)	25 (24.0)	0.109
Chronic kidney disease (GFR < 60 mL/min/1.73m^2^)	16 (13.0)	2 (10.5)	14 (13.5)	0.569
*Electrocardiography*				
PR interval, ms	187.8 ± 35.0	191.5 ± 44.9	187.1 ± 33.1	0.612
QRS duration, ms	168.5 ± 16.6	164.4 ± 17.9	169.2 ± 16.3	0.243
LBBB definition according to ESC 2013	108 (87.8)	15 (78.9)	93 (89.4)	0.199
CRT indication class				0.004*
I	100 (81.3)	11 (57.9)	89 (85.6)	
IIA	23 (18.7)	8 (42.1)	15 (14.4)	
*Echocardiography*				
LACI, %	18.0 [12.7–23.5]	20.9 [18.6–35.3]	16.5 [12.6–22.8]	0.015*
**LACI, quartile**				0.015*
Quartile 1	30 (24.4)	3 (15.8)	27 (26.0)	
Quartile 2	30 (24.4)	1 (5.3)	29 (27.9)	
Quartile 3	30 (24.4)	7 (36.8)	23 (22.1)	
Quartile 4	30 (24.4)	8 (42.1)	22 (21.2)	
LA EDVi, mL/m^2^	18.1 [13.0–24.5]	20.9 [16.1–28.5]	17.8 [11.9–23.7]	0.088
LA ESVi, mL/m^2^	31.2 [24.5–42.0]	37.0 [25.7–47.9]	30.1 [23.2–40.0]	0.067
LV EDVi, mL/m^2^	98.7 [79.2–122.9]	85.1 [67.1–111.3]	100.2 [80.4–126.2]	0.115
LV ESVi, mL/m^2^	71.5 [57.1–94.4]	62.0 [54.9–77.8]	73.3 [57.7–95.8]	0.111
LV ejection fraction, %	27.2 [19.4–31.5]	27.8 [21.6–31.0]	27.2 [19.3–31.6]	0.897
**Diastolic dysfunction**				0.707
Grade 1	76 (61.8; 76.0†)	12 (63.2; 80.0†)	64 (61.5; 75.3†)	
Grade 2	11 (8.9; 11.0†)	2 (10.5; 13.3†)	9 (8.7; 10.6†)	
Grade 3	13 (10.6; 13.0†)	1 (5.3; 6.7†)	12 (11.5; 14.1†)	
MV E/A ratio	0.66 [0.55–1.03]	0.89 [0.62–1.22]	0.64 [0.53–0.94]	0.103
E/e’ ratio	9.8 [7.3–13.2]	9.7 [6.9–15.7]	10.0 [7.3–12.9]	0.832
**Mitral regurgitation**				0.238
No to mild	94 (76.4)	15 (78.9)	79 (76.0)	
Moderate	25 (20.3)	4 (21.1)	21 (20.2)	
Severe	4 (3.3)	0 (0.0)	4 (3.8)	
TR max velocity, m/s	1.80 ± 0.90	1.81 ± 0.78	1.80 ± 0.93	0.957
Apical rocking	95 (77.2)	10 (52.6)	85 (81.7)	0.004*
Septal flash	87 (70.7)	10 (52.6)	77 (74.0)	0.050*
IVMD, ms	60 [40–80]	50 [20–75]	60 [40–80]	0.248

Categorical values are expressed as frequencies and percentages. †Indicate valid percentages excluding missing values; other percentages are based on the entire study sample. Normally distributed continuous values are presented as means ± SD; non-normally distributed values as medians [IQR]. *Indicates statistical significance. CMR = cardiac magnetic resonance; CRT = cardiac resynchronization therapy; EDVi = indexed end-diastolic volume; ESC = European Society of Cardiology; ESVi = indexed end-systolic volume; GFR glomerular filtration rate; IVMD = interventricular mechanical delay; IQR = interquartile range; LACI = left atrioventricular coupling index; LV = left ventricle; MV = mitral valve; NYHA = New York heart association; SD = standard deviation; TR = tricuspid regurgitation

## Results

### Study population

A total of 104 (85%) participants were classified as CRT responders, while 19 (15%) were identified as non-responders. The baseline characteristics of the study sample, including demographic, clinical, and echocardiographic characteristics, are presented in Table 1. Among the total 123 patients, 77 patients (63%) were male, mean age was 66 ± 10 years, and 49 (40%) patients had an ischemic cardiomyopathy. A larger percentage of responders had a Class I CRT indication (86% vs. 58%; *p* = 0.004). There were no significant differences in LA or LV volumes at baseline (all *p* > 0.05).

**Fig. 1:**
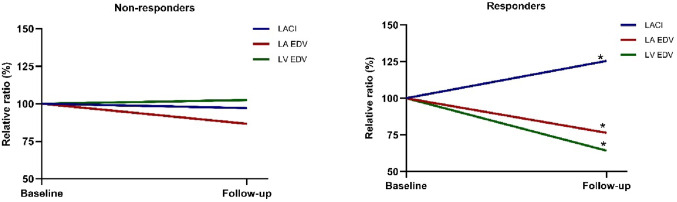
Changes in LACI, LA and LV end-diastolic volume after six months of CRT. *In non-responders (left)*,* no significant changes were observed in LACI*,* LA EDV*,* or LV EDV between baseline and follow-up. In responders (right)*,* LACI significantly increased*, while LA EDV and LV EDV significantly decreased. *Indicates statistical significance. EDV = end-diastolic volume; ESV = end-systolic volume; LA = left atrium; LACI = left atrioventricular coupling index; LV = left ventricle.

### Left atrial and ventricular volumes and LACI

In responders, a significant reduction in LA and LV EDVi was observed *(*Tables 1 and 2; Fig. 1). LA EDVi declined with 23.6% (17.8 [11.9–23.7] to 13.6 [10.2–20.4] mL/m²), while LV EDVi exhibited a more substantial decrease of 35.8% (100.2 [80.4–126.2.4.2] to 64.3 [48.8–76.4] mL/m²; both *p* < 0.001). In contrast, no significant changes in LA or LV EDVi were detected at follow-up in non-responders (both *p* > 0.05).

**Table 2:** Echocardiographic and electrocardiographic outcomes after six months of follow-up

	Total (*n* = 123)	Non-responders (*n* = 19)	Responders (*n* = 104)	*P*-value
LACI, %	20.7 [15.8–28.2]	20.3 [16.5–27.9]	20.7 [15.3–28.3]	0.794
LACI, quartile				0.275
Quartile 1	13 (10.6)	4 (21.1)	9 (8.7)	
Quartile 2	31 (25.2)	3 (15.8)	28 (26.9)	
Quartile 3	30 (24.4)	6 (31.6)	24 (23.1)	
Quartile 4	45 (36.6)	6 (31.6)	39 (37.5)	
LA EDVi, mL/m^2^	14.3 [11.2–20.7]	18.1 [15.1–24.3]	13.6 [10.2–20.4]	0.023*
LA ESVi, mL/m^2^	28.1 [19.4–34.6]	33.1 [28.5–40.1]	26.8 [18.3–33.9]	0.025*
LV EDVi, mL/m^2^	70.5 [53.0–82.7]	87.2 [79.0–99.7]	64.3 [48.8–76.4]	< 0.001*
LV ESVi, mL/m^2^	41.2 [28.5–55.2]	58.9 [50.3–74.9]	38.1 [27.5–50.5]	< 0.001*
LV ejection fraction, %	39.5 [31.4–45.9]	31.3 [24.9–35.8]	40.7 [32.9–46.7]	< 0.001*
Diastolic dysfunction				0.070
Grade 1	92 (74.8; 86.8†)	13 (68.4; 81.3†)	79 (76.0; 87.8†)	
Grade 2	11 (8.9; 10.4†)	1 (5.3; 6.3†)	10 (9.6; 11.1†)	
Grade 3	3 (2.4; 2.8†)	2 (10.5; 12.5†)	1 (1.0; 1.1†)	
MV E/A ratio	0.72 [0.55–0.92]	0.77 [0.50–1.06]	0.72 [0.56–0.90]	0.832
E/e’ ratio	9.4 [7.3–14.7]	9.6 [8.6–17.4]	9.2 [7.2–14.5]	0.252
Mitral regurgitation				0.175
No to mild	107 (87.0)	13 (68.4)	94 (90.4)	
Moderate	14 (11.4)	6 (31.6)	8 (7.7)	
Severe	1 (0.8)	0 (0.0)	1 (1.0)	
TR max velocity, m/s	1.62 ± 0.60	1.49 ± 0.62	1.64 ± 0.60	0.352
Apical rocking	36 (29.3)	7 (36.8)	29 (27.9)	0.430
Septal flash	11 (8.9)	4 (21.1)	7 (6.7)	0.046*
IVMD, ms	30 [5–50]	25 [0–40]	30 [7.5–50]	0.333
PR interval, ms	149.6 ± 23.9	148.0 ± 35.6	149.9 ± 21.6	0.813

Categorical values are expressed as frequencies and percentages. †Indicate valid percentages excluding missing values; other percentages are based on the entire study sample. Normally distributed continuous values are presented as means ± SD; non-normally distributed values as medians [IQR]. *Indicates statistical significance. EDVi = indexed end-diastolic volume; ESVi = indexed end-systolic volume; IVMD = interventricular mechanical delay; IQR = interquartile range; LACI = left atrioventricular coupling index; LV = left ventricle; MV = mitral valve; SD = standard deviation; TR = tricuspid regurgitation

In the entire study sample, median baseline LACI was 18.0% [12.7–23.5], without differences between males and females (*p* = 0.542). The first to fourth quartiles of LACI were ≤ 12.7%; 12.7% to 18.0%; 18.0% to 23.5%; and > 23.5%, respectively. Baseline LACI was lower in responders compared to non-responders (16.5% vs. 20.9%; *p* = 0.015). Six months after CRT implantation, LACI significantly increased to 20.7% in responders (*p* < 0.001*)*, whereas it remained stable in non-responders (20.9 vs. 20.3%; *p* = 0.077). As a result, LACI values at follow-up were similar between responders and non-responders (*p* = 0.794). The interobserver agreement for LACI measurements was excellent, with a two-way random-effects single-measures ICC of 0.90 (95% CI: 0.79–1.00).

**Fig. 2:**
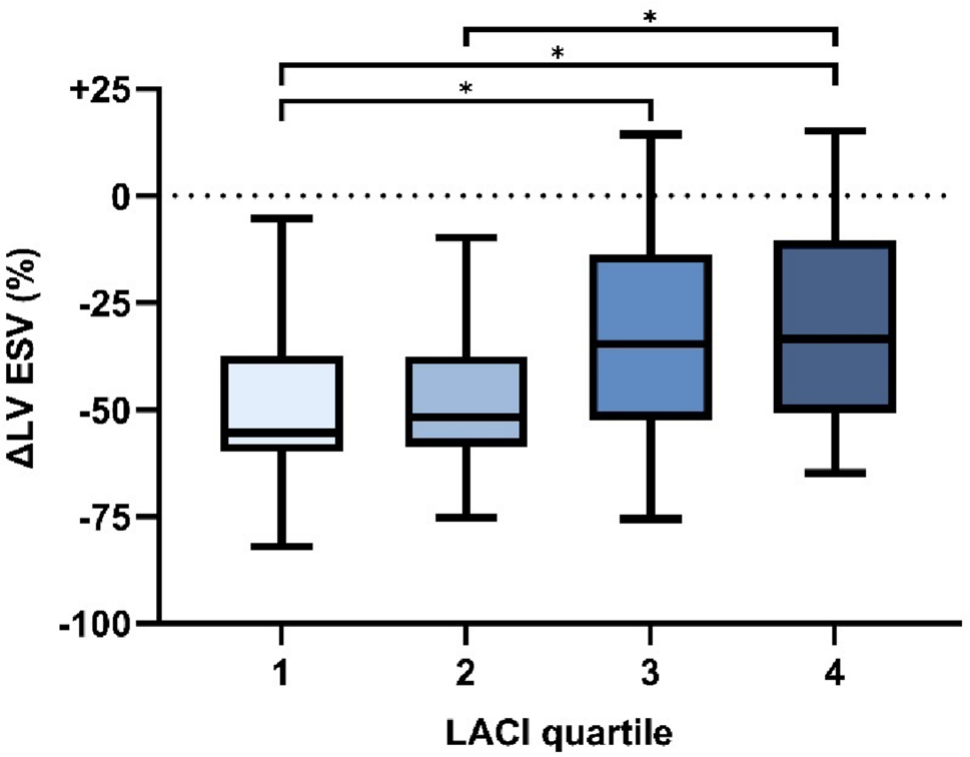
Correlation between baseline LACI and CRT response after six months. *Lower baseline LACI is associated with greater LV ESV reduction. CRT response varied across LACI quartiles, with significant differences observed between quartiles 1–3, 1–4, and 2–4. CRT = cardiac resynchronization therapy; ESV = end-systolic volume; LACI = left atrioventricular coupling index; LV = left ventricle*. *Indicates statistical significance.

### Prognostic value of LACI for CRT response

A significant correlation was observed between baseline LACI and LV reverse remodeling at six months (*p* = < 0.001; Fig. 2), with lower baseline LACI associated with greater LV ESVi reduction. The extent of reverse remodeling varied across LACI quartiles, with significant differences observed between quartiles 1–3, 1–4, and 2–4.

**Table 3:** Prognostic linear regression model performance for LVESVi change: LACI-only versus its components (LA and LV EDVi)

Model	Variables	Adjusted *R*^2^	SEE	*P*-value
LACI-only	LACI, %	0.138	20.914	< 0.001*
Components	LA EDVi + LV EDVi, mL/m^2^	0.057	21.881	0.013*

*Indicates statistical significance. Both models: *N* = 119.EDVi = indexed end-diastolic volume; ESVi = indexed end-systolic volume; LACI = left atrioventricular coupling index; LA = left atrium; LV = left ventricle; SEE = standard error of the estimate

LACI provided incremental prognostic value for LV reverse remodeling beyond all individual LA and LV volumes (ΔR² = 0.118–0.130; all incremental F-tests *p* < 0.001; Supplementary Table*S1*, with full coefficients and VIFs detailed in Supplementary Tables S2A*–*S2Dnull. The LACI-only model demonstrated better fit for reverse remodeling than the model including its components (LA + LV EDVi; adj. R² 0.138 vs. 0.057; both *p* < 0.05; Table 3).

**Table 4:** Univariable and multivariable linear regression analyses for percentage change in indexed LV end-systolic volume: full models

Univariable				Multivariable		
Variable	B	95% CI	*P*-value	B	95% CI	*P*-value
*Model 1: LACI modeled per 1% increase*						
LACI, %	0.808	0.450 to 1.167	< 0.001*	0.437	0.036 to 0.838	0.033*
Age, years	0.13	−0.305 to 0.565	0.554			
Female sex	−9.087	−17.357 to −0.818	0.032*	−2.268	−10.572 to 6.037	0.589
Non-ischemic cardiomyopathy	−13.178	−21.168 to −5.188	0.001*	−8.148	−16.654 to 0.358	0.060
Image-guided LV lead placement	−5.004	−13.171 to 3.162	0.227			
LV pacing electrode in target^†^	−7.411	−15.567 to 0.745	0.075			
LBBB^‡^	−8.711	−20.983 to 3.561	0.162			
Diastolic dysfunction grade	4.822	−1.164 to 10.808	0.113			
QRS duration/LV EDV, ms/mL	−25.042	−45.354 to −4.730	0.016*	−8.283	−27.251 to 10.686	0.388
Relative PR interval reduction after six months, %	0.013	−0.333 to 0.360	0.939			
Apical rocking	−20.202	−29.334 to −11.071	< 0.001*	−12.055	−22.547 to −1.562	0.025*
IVMD, ms	−0.209	−0.319 to −0.099	< 0.001*	−0.088	−0.209 to 0.032	0.148
*Model 2: Low (Q1-2) vs. high (Q3-4) LACI*						
LACI, low vs. High [ref]	−17.233	−24.821 to −9.645	< 0.001*	−11.163	−19.437 to −2.889	0.009*
Age, years	0.13	−0.305 to 0.565	0.554			
Female sex	−9.087	−17.357 to −0.818	0.032*	−1.686	−9.913 to 6.541	0.685
Non-ischemic cardiomyopathy	−13.178	−21.168 to −5.188	0.001*	−9.274	−17.645 to −0.904	0.035*
Image-guided LV lead placement^†^	−5.004	−13.171 to 3.162	0.227			
LV pacing electrode in target	−7.411	−15.567 to 0.745	0.075			
LBBB^‡^	−8.711	−20.983 to 3.561	0.162			
Diastolic dysfunction grade	4.822	−1.164 to 10.808	0.113			
QRS duration/LV EDV, ms/mL	−25.042	−45.354 to −4.730	0.016*	−8.362	−27.074 to 10.350	0.377
Relative PR interval reduction after six months, %	0.013	−0.333 to 0.360	0.939			
Apical rocking	−20.202	−29.334 to −11.071	< 0.001*	−12.471	−22.731 to −2.211	0.018*
IVMD, ms	−0.209	−0.319 to −0.099	< 0.001*	−0.067	−0.188 to 0.054	0.274

Covariates associated with ΔLV ESVi at a significance level of *P* < 0.05 in univariable analyses were included in the multivariable linear regression model. *Indicates statistical significance. † Any of the three scar-free segments with latest mechanical activation as determined using CARTBox-Suite (V3.1, CARTTech BV). ‡ Definition according to ESC 2013. CI = confidence interval; EDV(i) = (indexed) end-diastolic volume; ESC = European society of cardiology; ESV(i) = (indexed) end-systolic volume; IVMD = interventricular mechanical delay; LACI = left atrioventricular coupling index; LA = left atrium; LBBB = left bundle branch block; LV = left ventricle

Two full linear regression models were constructed: Model 1 included LACI as a continuous variable, and Model 2 used LACI dichotomized into low (Q1–2) and high (Q3–4) categories (Table 4). Univariable predictors of ΔLV ESVi included LACI, female sex, non-ischemic cardiomyopathy, QRS duration standardized to LV volume, apical rocking, and interventricular mechanical delay (IVMD). LACI and apical rocking were consistently associated with LV reverse remodeling across both multivariable models, while non-ischemic cardiomyopathy reached significance only in the dichotomized model (Model 2). In Model 1, each 1–percentage-point increase in LACI was associated with a 0.44% smaller reduction in LV ESVi (95% CI: 0.04 to 0.84; *p* = 0.033). In Model 2, patients with low LACI values (Q1-2) experienced an 11.2% greater reduction in LV ESVi compared to those in with high LACI values (Q3-4) (95% CI: − 19.44 to − 2.89; *p* = 0.009).

In univariable logistic regression, LACI, QRS duration standardized to LV volume, and apical rocking were significantly associated with CRT response (Supplementary Table S3). In multivariable analysis, only LACI retained statistical significance, with low LACI associated with a 3.63-fold higher odds of CRT response compared to high LACI (95% CI: 1.05–12.55; *p* = 0.041). Each 1–percentage-point increase in baseline LACI was significantly associated with 5.1% lower odds of CRT response (OR: 0.95; 95% CI: 0.91–0.99; *p* = 0.028).

**Fig. 3:**
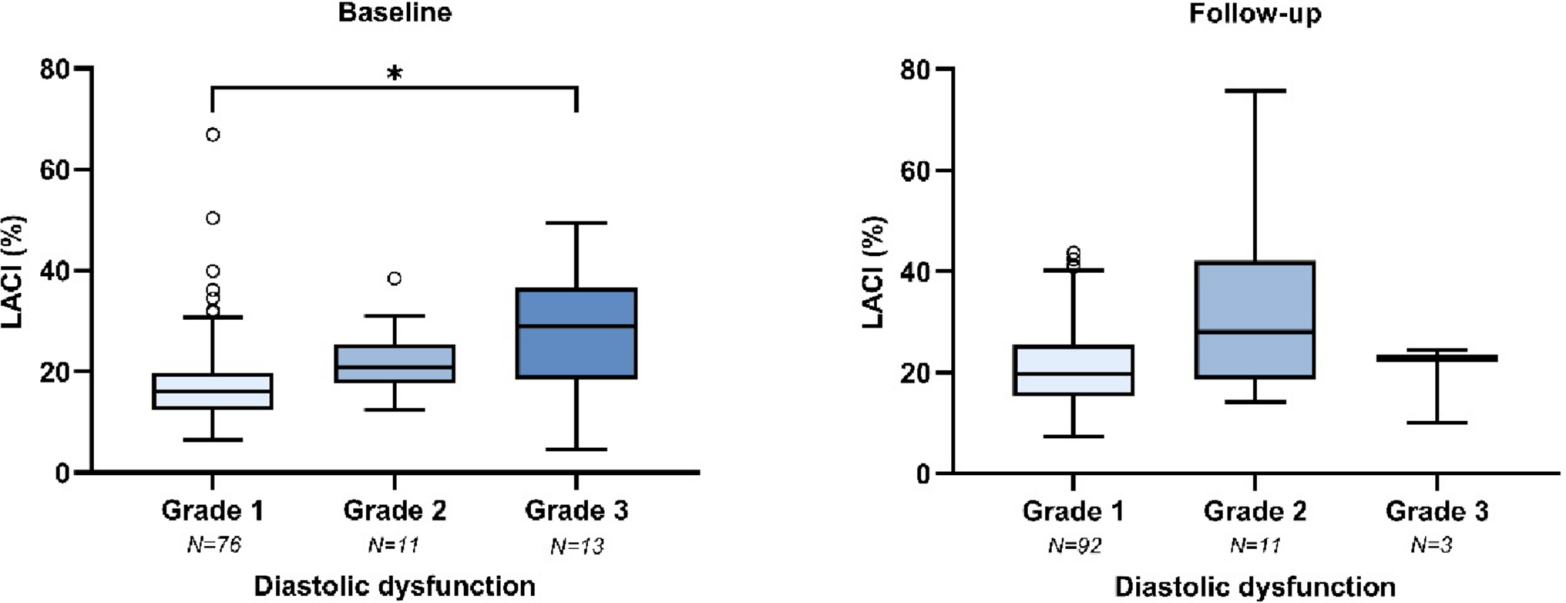
Correlation between LACI and diastolic dysfunction grade at baseline and follow-up. *At baseline (left)*,* LACI differed significantly between grade 1 and grade 3 at baseline. At follow-up (right)*, no significant differences were observed. Patients with missing LACI or diastolic function data were excluded from the analysis. *Indicates statistical significance

### Correlation with diastolic function

The majority of the study population (76%) was classified with grade 1 diastolic dysfunction at baseline, while 11% were categorized as grade 2 and 13% as grade 3 diastolic dysfunction. After six months of CRT, diastolic dysfunction severity decreased by 0.3 ± 0.7 grades in responders (*p* = 0.003), whereas non-responders showed no significant change (+ 0.1 ± 0.5 grades; *p* = 0.564). The proportion of patients with grade 1 diastolic dysfunction increased to 87%, and the remaining patients were classified with grade 2 (10%) or grade 3 (3%) diastolic dysfunction. Supplementary Figure *S1* illustrates the transitions in diastolic dysfunction gradation from baseline to follow-up. Greater diastolic impairment was associated with higher baseline LACI values, with a significant difference observed between grade 1 and grade 3 diastolic dysfunction (Fig. 3). Analysis of separate diastolic function parameters showed significant associations between LACI and MV E/A ratio, E/e’ ratio, LA ESVi, and tricuspid regurgitation velocity *(*Supplementary Table S4*).* At follow-up, no significant differences in LACI were observed across diastolic dysfunction grades. Supplementary Figure S2 illustrates the correlations between LACI and LA ESVi at baseline and follow-up.

### Correlation with functional class

At baseline, 72% of patients were in NYHA class II, while 2% and 25% were classified as class I and III, respectively. After six months of CRT, NYHA class significantly improved in responders, with a mean change of − 0.5 ± 0.6 classes (*p* < 0.001). In non-responders, the change in NYHA class was not statistically significant (–0.5 ± 0.9; *p* = 0.052). Transitions in NYHA class between baseline and follow-up are illustrated in Supplementary Figure S3. No significant association was observed between baseline LACI and change in NYHA class (ρ = 0.131; *p* = 0.208). Supplementary Figure S4 presents boxplots of baseline LACI according to the degree of NYHA class change after six months.

### Adverse events

A total number of seven heart failure-related adverse events occurred during a median follow-up period of 19.7 [13.5–25.7] months. An overview of the events is provided in Supplementary Table S5*.* No significant association between baseline LACI and heart failure-related adverse events was observed (*p =* 0.107).

## Discussion

The present study is the first to evaluate the novel LACI metric in CRT recipients. Our main findings are: (1) LACI is independently associated with CRT response; (2) LACI increases in responders due to more pronounced LV than LA reverse remodeling, precluding its use for AV coupling assessment; and (3) LACI demonstrates excellent interobserver reliability.

### Low LACI is independently associated with echocardiographic CRT response

In line with previous research, we observed that CRT induces significant LA and LV remodeling, albeit exclusively in responders (Fig. 1) [13, 14]. Baseline LACI showed an inverse association with LV reverse remodeling (Fig. 2). Lower LACI values may arise from a relatively small LA EDV, a larger LV EDV, or both. Consistently, prior studies have demonstrated that smaller LA volumes [13, 28], reduced LA diameters [29], normal LA pressures [30], and preserved LA function [31, 32], features typically indicative of lower filling pressures, are associated with beneficial CRT outcomes. Likewise, a dilated LV has been linked to greater LV reverse remodeling potential [33, 34]. In our cohort, LACI was more informative than all individual LA and LV volume indices for CRT response, and the LACI-only model demonstrated superior fit versus the model including LA and LV EDVi, indicating incremental value of the LA: LV ratio (Table 3; Supplementary Tables S1–S2). In linear and logistic multivariable regression analyses, LACI remained independently associated with CRT response. Taken together, low LACI may denote a structural substrate characterized by a relatively undiseased LA alongside a dilated, dyssynchronous LV, with greater remodeling reserve, less irreversible myocardial injury, and milder degree of diastolic dysfunction.

### LACI does not reflect AV coupling after CRT

CRT responders exhibited lower baseline LACI values compared to non-responders. Based on the assumption that CRT restores AV synchrony [5–8, 11], a reduction in LACI was anticipated in responders, as lower values are generally indicative of more favorable AV coupling. In contrast, an increase in LACI was observed in responders, reaching values comparable to those of non-responders, in whom LACI remained stable. The paradoxical LACI increase in responders was driven by more pronounced LV than LA reverse remodeling, thereby concealing any improvement in AV synchrony which would typically lower LACI (Fig. 1). Consequently, LACI is unsuitable for assessing AV coupling post-CRT.

### High reliability of LACI measurements

Our analysis confirmed excellent interobserver reliability for LACI measurements (ICC = 0.90; 95% CI: 0.79–1.00), demonstrating strong consistency and reproducibility. These findings are consistent with prior studies, which reported interobserver ICC values of 0.81 (95% CI: 0.71–0.88) for CMR and 0.95 (95% CI: 0.81–0.99) for three-dimensional echocardiography [18, 19].

### LACI reflects diastolic dysfunction severity pre-CRT

Consistent with a prior study in heart failure patients [20], we observed a positive correlation between pre-CRT LACI and diastolic dysfunction severity, albeit with lower LACI values across dysfunction grades *(*Fig. 3*).* This difference may be explained by a higher proportion of patients with grade 1 diastolic dysfunction in our cohort (76% vs. 41%), lower baseline LA ESVi (18 vs. 29 mL/m^2^), and fewer cases of severe mitral regurgitation (3% vs. 12%). The observed correlation likely reflects elevated LV filling pressures leading to increased LA pressure, LA dilatation, and consequently, higher LACI values. Supporting this, LACI demonstrated a statistically significant moderate correlation with LA ESVi and several other conventional diastolic function parameters (Supplementary Table S4; Supplementary Figure S2*).*

At follow-up, no significant differences in LACI were observed across diastolic dysfunction grades. This attenuation of the association may be attributed to the small proportion of patients with severe dysfunction in our study sample. At baseline, 13% of patients were classified with grade 3 diastolic dysfunction. After six months, this proportion decreased to 3%, in stark contrast to the 26% reported in the previous study [20]. Furthermore, CRT-induced increase in LACI, resulting from greater LV than LA reverse remodeling, may have masked any residual association between LACI and diastolic dysfunction at follow-up. Nonetheless, LA ESVi remained significantly correlated with LACI at follow-up (Supplementary Figure S2). LACI may thus serve as a rapid and practical metric for estimating diastolic function, although in our cohort its reliability was limited to the pre-CRT setting.

### LACI is associated with structural but not symptomatic CRT response

While baseline LACI was associated with echocardiographic CRT response and NYHA class improved in responders, no significant association was found between LACI and change in functional class at six months follow-up (Supplementary Figure S4). This discordance may reflect the multifactorial and subjective nature of NYHA classification, which encompasses not only cardiac function but also comorbidities, physical conditioning, and individual symptom perception. LACI serves as a surrogate structural marker of AV coupling. Our findings suggest that while LACI is valuable in predicting objective reverse remodeling, it may not directly translate to perceived symptomatic improvement. Alternatively, the high proportion of NYHA class II patients (72%) at baseline and the strong CRT response rate (85%) may have reduced the variability in NYHA class improvement, thereby limiting statistical power.

### Limitations

Several limitations of this study should be acknowledged. First, LA and LV volumes were assessed using echocardiography, whereas most previous studies on LACI employed CMR, which remains the reference standard for volume quantification [18, 35–38]. Although CMR data were available for all participants, the image acquisition protocol of the ADVISE-CRT III study primarily included contiguous short-axis cine imaging [22]. Two- and four-chamber long-axis cine views required for biplane LA volume assessment were not uniformly available, precluding robust analyses using CMR-derived LACI [39]. In addition, echocardiographic strain analysis was not included in the ADVISE-CRT III study protocol, and suboptimal image quality precluded retrospective strain assessment. Comparisons between LACI and strain-based metrics could consequently not be performed. While echocardiography is subject to limitations such as acoustic window dependency, its broad availability, lower cost, and standard use in CRT evaluation support its clinical applicability for LACI measurement.

Another limitation is the high proportion of patients with LBBB morphology (88%), limiting generalizability of our findings to non-LBBB patients. In LBBB patients, the primary mechanism of benefit is ventricular resynchronization. In patients with non-LBBB morphology however, the extent to which ventricular resynchronization can be achieved remains uncertain [3, 4]. In these cases, alternative mechanisms, such as the restoration of AV coupling, may play a more prominent role and influence LACI after CRT implantation. Nonetheless, this study provides the first evaluation of LACI in CRT recipients and offers important preliminary insights into its clinical utility. Given that the majority of patients in the present cohort had a Class I indication, the findings are relevant to most contemporary CRT candidates.

Furthermore, the ADVISE-CRT III study was primarily designed to evaluate in-target LV lead placement with image guidance, and the sample size calculation was based on this outcome [22]. As shown in Fig. 3, LACI tended to increase with advancing diastolic dysfunction. However, the limited number of patients with grade 2 or 3 dysfunction reduced the statistical power to detect differences across all grades. Still, a significant difference in baseline LACI was observed between grade 1 and grade 3 diastolic dysfunction. Similarly, no associations were found between LACI and either functional improvement or heart failure-related adverse events. These analyses may have been underpowered due to the predominance of NYHA class II patients (72%) and the low number of adverse events (*n* = 7) during follow-up.

Lastly, our study sample demonstrated an exceptionally high CRT response rate, with 85% of patients classified as responders and a median LV ESVi reduction of 44% [26–56], consistent with a super-responder profile. This disproportionate distribution limited the feasibility of performing a robust receiver operating characteristic (ROC) analysis to determine an optimal LACI cutoff value. Nonetheless, LACI was independently associated with CRT response in both multivariable linear and logistic regression models, supporting its prognostic utility (Table 4*and *Supplementary Table S3). It is conceivable that the predictive effect of LACI may be more pronounced in a cohort with a broader distribution of response.

## Conclusion

Lower LACI is independently associated with echocardiographic CRT response and provides incremental prognostic value beyond individual LA and LV volumetric measures. Six months post-implantation, CRT responders demonstrated an increase in LACI, driven by a disproportionately greater LV compared to LA reverse remodeling. This remodeling imbalance may obscure any CRT-mediated improvement in AV coupling, which would typically lower LACI. Hence, while LACI may capture favorable structural substrate for CRT and AV coupling at baseline, it does not reliably reflect AV coupling after CRT.

## Supplementary Information

Below is the link to the electronic supplementary material.

Supplementary Material 1

## Data Availability

No datasets were generated or analysed during the current study.

## Abbreviations

ADVISE-CRT III: Advanced Image Supported Left Ventricular Lead Placement in Cardiac Resynchronization Therapy
ASE: American society of echocardiography
AV: Atrioventricular
BSA: Body surface area
CMR: Cardiac magnetic resonance imaging
CRT: Cardiac resynchronization therapy
CT: Computed tomography
EACVI: European association of cardiovascular imaging
ECG: Electrocardiogram
EDV(i): End-diastolic volume (indexed to BSA)
ESC: European society of cardiology
ESV(i): End-systolic volume (indexed to BSA)
ICC: IntraClass correlation coefficient
IVMD: interventricular mechanical delay
LA: Left atrium
LACI: Left atrioventricular coupling index
LBBB: Left bundle branch block
LV: Left ventricle
NYHA: New York heart association
ROC: Receiver operating characteristic
VIF: Variance inflation factor
