# Supplementary material for: Left atrioventricular coupling index in patients undergoing cardiac resynchronization therapy
Source: Int J Cardiovasc Imaging. 2026 Feb 14;42(5):939–51. doi: 10.1007/s10554-026-03641-9 (PMC13136192; doi:10.1007/s10554-026-03641-9)
Supplement: Supplementary file 1 — Supplementary Material 1 [file 10554_2026_3641_MOESM1_ESM.docx]

**
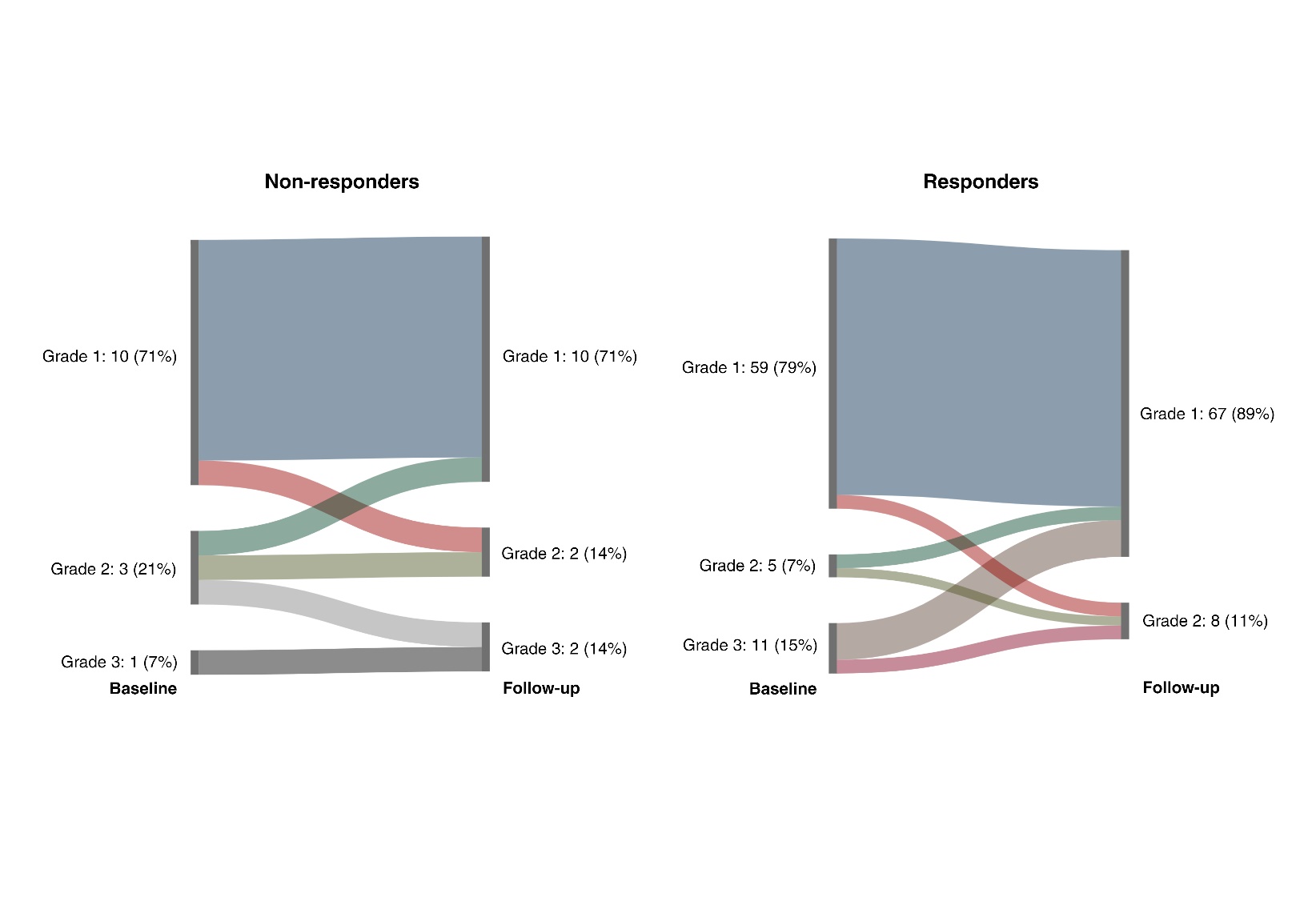
SUPPLEMENTARY
Supplementary Figure S1. Diastolic dysfunction grade transition between baseline and follow-up in non-responders and responders.***The left Sankey diagram visualizes the transition of diastolic dysfunction grades in non-responders, while the right diagram illustrates these transitions in responders. Only patients with diastolic function grades recorded at both baseline and follow-up time points were included in this analysis. Percentages might not add up to 100% due to rounding.*


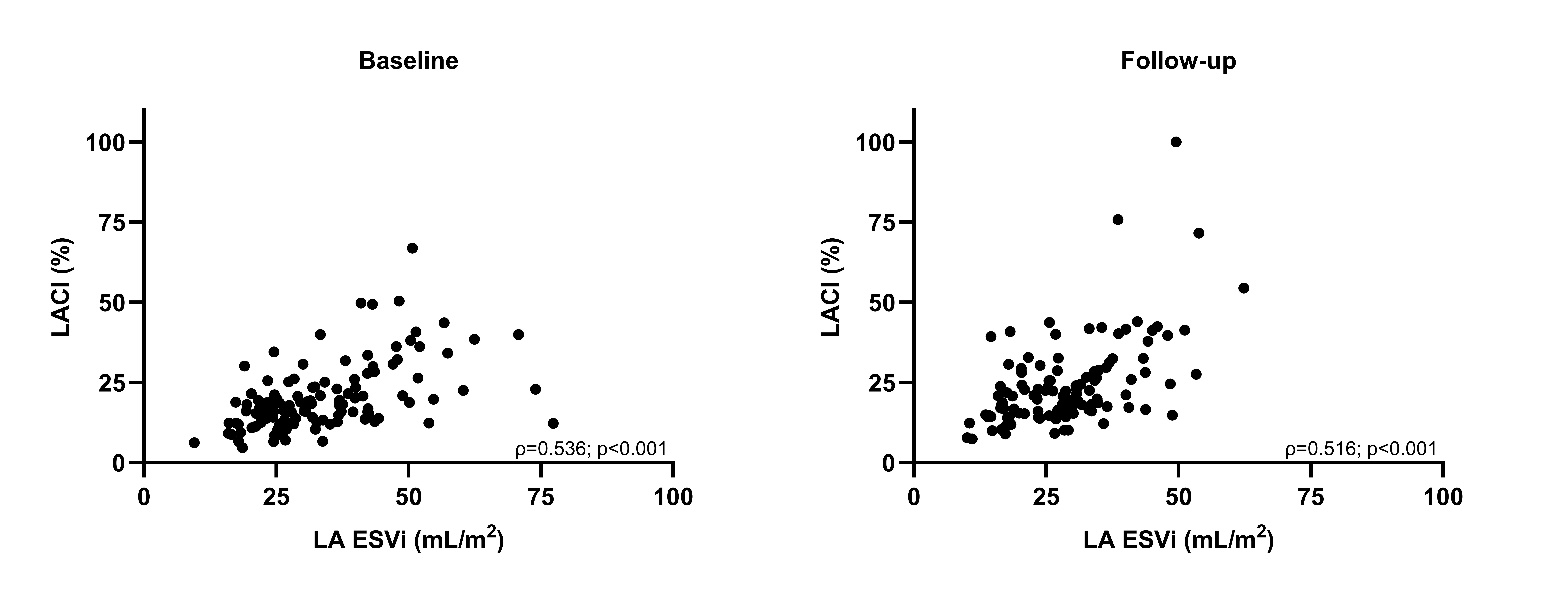
**Supplementary Figure S2. Correlation between LACI and LA ESVi at baseline and follow-up.***A positive correlation between LACI and LA ESVi was observed both at baseline and after six months of follow-up.*
*ρ = Spearmans’ rho; LA = left atrium; ESVi = indexed end-systolic volume; LACI = left atrioventricular coupling index.*

**
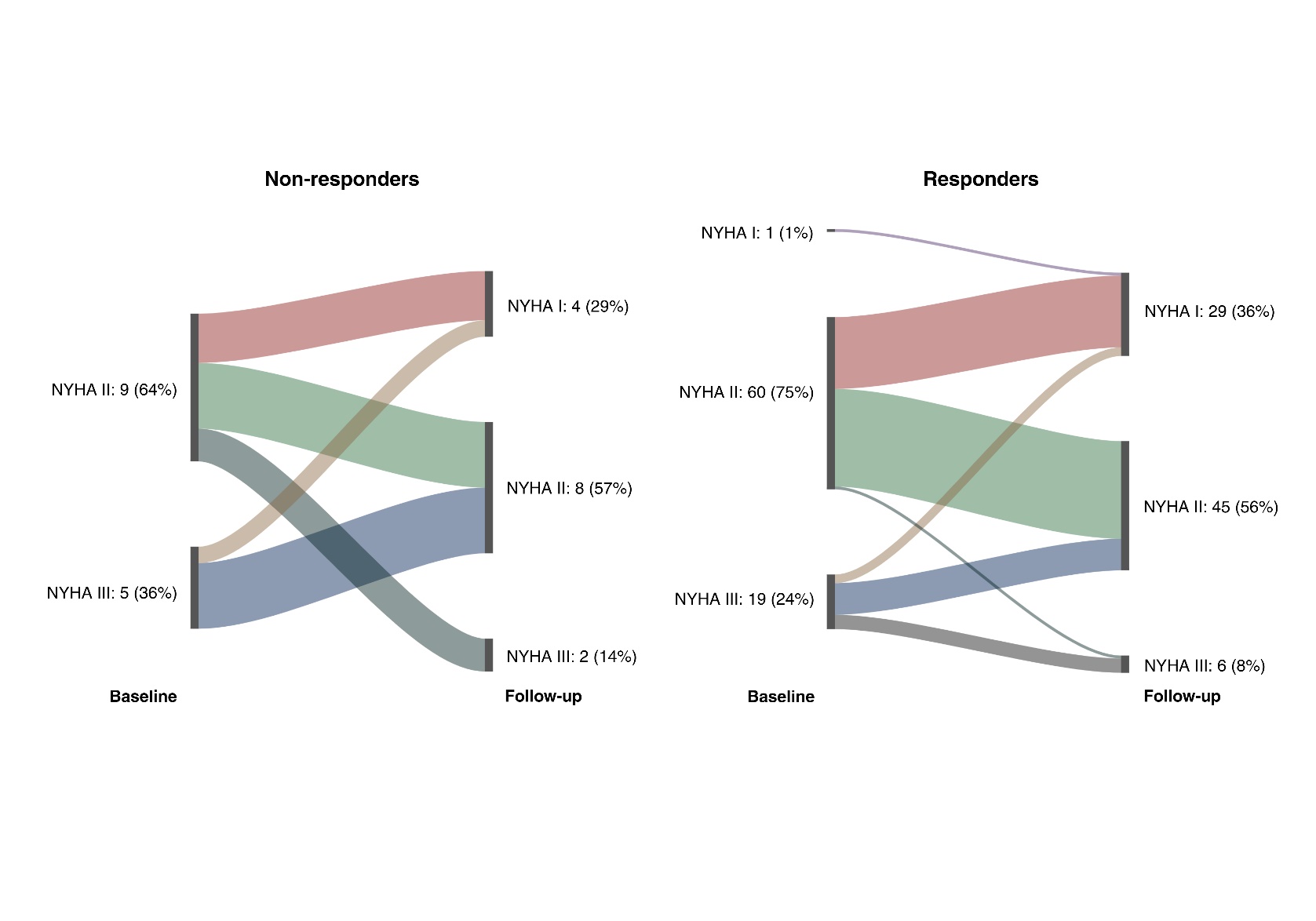
Supplementary Figure S3. NYHA class transition between baseline and follow-up in non-responders and responders.***The left Sankey diagram visualizes the transition of NYHA class in non-responders, while the right diagram illustrates these transitions in responders. Only patients with available NYHA classifications at both baseline and follow-up were included in the analysis.
NYHA = New York Heart Association.*

**
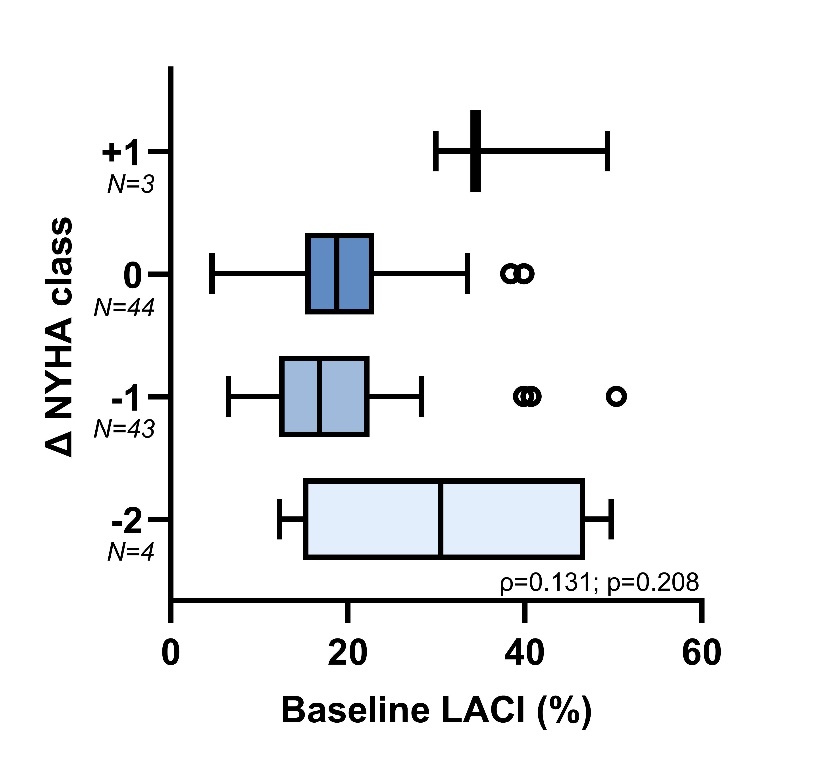
**

**Supplementary Figure S4. Association between LACI and NYHA class change.***Boxplots depicting baseline LACI according to change in NYHA class from baseline to six months follow-up. No significant association was observed. Only patients with available NYHA classifications at both baseline and follow-up were included in the analysis.
ρ = Spearmans’ rho; LACI = left atrioventricular coupling index; NYHA = New York Heart Association.*

**Supplementary Table S1. Incremental prognostic value of LACI beyond individual LA and LV volumes for LV ESVi change (ΔR² and F-change)**

| **Base** | **R^2^ (base)** | **R^2^ (base + LACI)** | **ΔR²** | **F change  (base + LACI)** | **Significant F change  (base + LACI)** |
| --- | --- | --- | --- | --- | --- |
| LA EDVi, mL/m^2^ | 0.033 | 0.160 | 0.128 | 17.630 | <0.001* |
| LA ESVi, mL/m^2^ | 0.021 | 0.135 | 0.129 | 17.570 | <0.001* |
| LV EDVi, mL/m^2^ | 0.016 | 0.131 | 0.130 | 17.611 | <0.001* |
| LV ESVi, mL/m^2^ | 0.037 | 0.155 | 0.118 | 16.228 | <0.001* |

*Reported values are unadjusted. Base refers to the model with the respective volumetric parameter alone; base + LACI refers to the combined model. ΔR² indicates the change in explained variance when LACI was added to the base model. F change refers to the incremental F test of model improvement. All models: N=119.
*Indicates statistical significance. All VIFs for combined models < 2.
EDVi = indexed end-diastolic volume; ESVi = indexed end-systolic volume; LACI = left atrioventricular coupling index; LA = left atrium; LV = left ventricle; VIF = variance inflation factor.*

**Supplementary Table S2A. Incremental prognostic value of LACI beyond LA EDVi for LV ESVi percentage change (coefficients)**

|  | **Base model** |  |  | **Base + LACI** |  |  |  |
| --- | --- | --- | --- | --- | --- | --- | --- |
| **Variable** | **B** | **95% CI** | ***P*-value** | **B** | **95% CI** | ***P*-value** | **VIF** |
| LA EDVi, mL/m^2^ | 0.409 | 0.002 to 0.817 | 0.049* | -0.387 | -0.927 to 0.148 | 0.154 | 1.973 |
| LACI, % | - | - | - | 1.063 | 0.562 to 1.564 | <0.001* | 1.973 |

*R² 0.033 → 0.160; ΔR² 0.128; ΔF (1,116) = 17.630; p < 0.001.
* Indicates statistical significance. Reported values are unadjusted.
CI = confidence interval; EDVi = indexed end-diastolic volume; LACI = left atrioventricular coupling index; LA = left atrium; VIF = variance inflation factor.*

**Supplementary Table S2B. Incremental prognostic value of LACI beyond LA ESVi for LV ESVi percentage change (coefficients)**

|  | **Base model** |  |  | **Base + LACI** |  |  |  |
| --- | --- | --- | --- | --- | --- | --- | --- |
| **Variable** | **B** | **95% CI** | ***P*-value** | **B** | **95% CI** | ***P*-value** | **VIF** |
| LA ESVi, mL/m^2^ | 0.252 | -0.064 to 0.567 | 0.117 | -0.132 | -0.478 to 0.215 | 0.452 | 1.377 |
| LACI, % | - | - | - | 0.892 | 0.471 to 1.314 | <0.001* | 1.377 |

*R² 0.021 → 0.150; ΔR² 0.129; ΔF (1,116) = 17.570; p < 0.001.
* Indicates statistical significance. Reported values are unadjusted.
CI = confidence interval; ESVi = indexed end-systolic volume; LACI = left atrioventricular coupling index; LA = left atrium; VIF = variance inflation factor.*

**Supplementary Table S2C. Incremental prognostic value of LACI beyond LV EDVi for LV ESVi percentage change (coefficients)**

|  | **Base model** |  |  | **Base + LACI** |  |  |  |
| --- | --- | --- | --- | --- | --- | --- | --- |
| **Variable** | **B** | **95% CI** | ***P*-value** | **B** | **95% CI** | ***P*-value** | **VIF** |
| LV EDVi, mL/m^2^ | -0.057 | -0.140 to 0.025 | 0.171 | -0.006 | -0.087 to 0.075 | 0.884 | 1.099 |
| LACI, % | - | - | - | 0.800 | 0.422 to 1.177 | <0.001* | 1.099 |

*R² 0.016 → 0.146; ΔR² 0.130; ΔF (1,116) = 17.611; p < 0.001.
* Indicates statistical significance. Reported values are unadjusted.
CI = confidence interval; EDVi = indexed end-diastolic volume; LACI = left atrioventricular coupling index; LV = left ventricle; VIF = variance inflation factor.*

**Supplementary Table S2D. Incremental prognostic value of LACI beyond LV ESVi for LV ESVi percentage change (coefficients)**

|  | **Base model** |  |  | **Base + LACI** |  |  |  |
| --- | --- | --- | --- | --- | --- | --- | --- |
| **Variable** | **B** | **95% CI** | ***P*-value** | **B** | **95% CI** | ***P*-value** | **VIF** |
| LV ESVi, mL/m^2^ | -0.121 | -0.234 to -0.008 | 0.037* | -0.064 | -0.174 to 0.047 | 0.255 | 1.070 |
| LACI, % | - | - | - | 0.753 | 0.383 to 1.124 | <0.001* | 1.070 |

*R² 0.037 → 0.155; ΔR² 0.118; ΔF (1,116) = 16.228; p < 0.001.
* Indicates statistical significance. Reported values are unadjusted.
CI = confidence interval; ESVi = indexed end-systolic volume; LACI = left atrioventricular coupling index; LV = left ventricle; VIF = variance inflation factor.*

**Supplementary Table S3. Univariable and multivariable logistic regression analyses for CRT response.**

|  | **Univariable** |  | **Multivariable** |  |
| --- | --- | --- | --- | --- |
| **Variable** | **Odds ratio (95% CI)** | ***P*-value** | **Odds ratio (95% CI)** | ***P*-value** |
| *Model 1: LACI modeled per 1% increase* | | | | |
| LACI, % | 0.936 (0.896 to 0.977) | 0.003* | 0.949 (0.905 to 0.994) | 0.028* |
| Age, years | 0.999 (0.948 to 1.052) | 0.961 |  |  |
| Female sex | 1.974 (0.662 to 5.889) | 0.233 |  |  |
| Non-ischemic cardiomyopathy | 2.316 (0.856 to 6.265) | 0.098 |  |  |
| Image-guided LV lead placement | 2.432 (0.859 to 6.888) | 0.094 |  |  |
| LV pacing electrode in target^†^ | 1.248 (0.464-3.556) | 0.661 |  |  |
| LBBB^‡^ | 2.255 (0.635 to 8.009) | 0.209 |  |  |
| Diastolic dysfunction grade |  |  |  |  |
| Grade 1 vs. Grade 2-3 [ref] | 0.762 (0.196 to 2.962) | 0.695 |  |  |
| QRS duration/LV EDV, ms/mL | 24.661 (1.163 to 522.809) | 0.040* | 28.565 (0.875 to 932.474) | 0.059 |
| Relative PR interval reduction after six months, % | 2.047 (0.029 to 145.320) | 0.742 |  |  |
| Apical rocking | 4.250 (1.511 to 11.955) | 0.006* | 2.946 (0.938 to 9.251) | 0.064 |
| IVMD, ms | 1.010 (0.994 to 1.026) | 0.207 |  |  |
| *Model 2: Low (Q1-2) vs. high (Q3-4) LACI* | | | |  |
| LACI, low vs. high [ref] | 4.667 (1.447 to 15.046) | 0.010* | 3.634 (1.052 to 12.549) | 0.041* |
| Age, years | 0.999 (0.948 to 1.052) | 0.961 |  |  |
| Female sex | 1.974 (0.662 to 5.889) | 0.233 |  |  |
| Non-ischemic cardiomyopathy | 2.316 (0.856 to 6.265) | 0.098 |  |  |
| Image-guided LV lead placement | 2.432 (0.859 to 6.888) | 0.094 |  |  |
| LV pacing electrode in target^†^ | 1.248 (0.464-3.556) | 0.661 |  |  |
| LBBB^‡^ | 2.255 (0.635 to 8.009) | 0.209 |  |  |
| Diastolic dysfunction grade |  |  |  |  |
| Grade 1 vs. Grade 2-3 [ref] | 0.762 (0.196 to 2.962) | 0.695 |  |  |
| QRS duration/LV EDV, ms/mL | 24.661 (1.163 to 522.809) | 0.040* | 26.142 (0.805 to 849.032) | 0.066 |
| Relative PR interval reduction after six months, % | 2.047 (0.029 to 145.320) | 0.742 |  |  |
| Apical rocking | 4.250 (1.511 to 11.955) | 0.006* | 3.055 (0.983 to 9.493) | 0.054 |
| IVMD, ms | 1.010 (0.994 to 1.026) | 0.207 |  |  |

*Covariates associated with CRT response at a significance level of P < 0.05 in univariable analyses were included in the multivariable linear regression model.
*Indicates statistical significance.
† Any of the three scar-free segments with latest mechanical activation as determined using CARTBox-Suite (V3.1, CARTTech BV). ‡ Definition according to ESC 2013.
CI = confidence interval; EDV(i) = (indexed) end-diastolic volume; ESC = European society of cardiology; ESV(i) = (indexed) end-systolic volume; IVMD = interventricular mechanical delay; LACI = left atrioventricular coupling index; LA = left atrium; LBBB = left bundle branch block; LV = left ventricle.*

**Supplementary Table S4. Correlation between LACI and diastolic function parameters at baseline and at follow-up.**

| **Baseline** | **Coefficient** | ***P*-value** |
| --- | --- | --- |
| Diastolic dysfunction grade | 0.424 | <0.001* |
| MV E/A ratio | 0.389 | <0.001* |
| E/e’ ratio | 0.229 | 0.029* |
| LA ESVi, mL/m^2^ | 0.536 | <0.001* |
| TR max velocity, m/s | 0.223 | 0.034* |
|  |  |  |

| **Follow-up** | **Coefficient** | ***P*-value** |
| --- | --- | --- |
| Diastolic dysfunction grade | 0.089 | 0.366 |
| MV E/A ratio | 0.123 | 0.206 |
| E/e’ ratio | 0.170 | 0.170 |
| LA ESVi, mL/m^2^ | 0.516 | <0.001* |
| TR max velocity, m/s | 0.122 | 0.211 |
|  |  |  |

********Indicates statistical significance.
ESVi = indexed end-systolic volume; LA = left atrium; MV = mitral valve; TR = tricuspid regurgitation.*

| **AEs related to heart failure** | **Frequency** |
| --- | --- |
| Orthostatic hypotension | 1 |
| LV thrombus | 1 |
| Non-sustained VT | 1 |
| Worsening dyspnea | 1 |
| **Total** | 4 |

**Supplementary Table S5. Overview of (severe) adverse events.**

| **SAEs related to heart failure** | **Frequency** |
| --- | --- |
| Slow VT requiring medical intervention | 2 |
| Decompensated heart failure resulting in death | 1 |
| **Total** | 3 |

*AE = adverse event; LV = left ventricle; LA = left atrium; SAE = severe adverse event.*
